# Born to run? Racing and training outcomes, population dynamics and traceability of a Thoroughbred birth cohort

**DOI:** 10.1002/vetr.5777

**Published:** 2025-09-26

**Authors:** Rebecca Mouncey, Amanda M. de Mestre, Kristien L. Verheyen

**Affiliations:** ^1^ Department of Pathobiology and Population Sciences Royal Veterinary College Hatfield UK; ^2^ Baker Institute for Animal Health College of Veterinary Medicine Cornell University Ithaca New York USA

**Keywords:** equine, racing, Thoroughbred, traceability, training, wastage

## Abstract

**Background:**

Analyses of industry‐level data suggest that around one‐third of the UK and Ireland Thoroughbred foal crop fail to enter training. Prospective follow‐up of individual horses could provide additional insight, particularly around individuals not attaining specific career milestones.

**Methods:**

A Thoroughbred birth cohort was established on stud farms across the UK and Ireland. Training, race performance, sales, export data, destinations and reasons for individuals failing to meet career milestones were collected from stud records, follow‐up with owners, stud book and racing authorities’ databases, and publicly available data sources to the end of the fourth year of life.

**Results:**

Of 262 foals that were alive at the end of the second year of life, 94.7% (248/262; 95% confidence interval [CI] 91.2‒96.8) were registered with a licensed trainer and 79.0% (207/262; 95% CI 73.7‒83.5) had raced at least once by the end of the follow‐up period. Just eight individuals, all of which had been sold as foals and/or yearlings, had an unknown fate or whereabouts.

**Limitations:**

Outcomes for horses exported out of the UK may be underestimated.

**Conclusions:**

Wastage may be lower than industry‐level figures suggest, highlighting important traceability gaps at this level, likely due to the dynamic nature of Thoroughbred populations.

## INTRODUCTION

Figures from analyses of industry‐level data and older studies suggest that the proportions of Thoroughbreds entering training or appearing on a racecourse seem to have remained largely unchanged over the last two decades, with around 60% of the foal crop entering training and around 40% racing at least once.^1^, ^2^, ^3^ Unfortunately, these studies^1^, ^2^, ^3^ provide very little explanation of the fate or whereabouts of the majority of individuals failing to attain these milestones, which has led to speculation around wastage and raised important welfare concerns,^4^ in turn threatening the industry's social licence to operate. The Horse Welfare Board's strategic plan for the welfare of horses bred for racing highlights the need to better understand the health and fate of Thoroughbreds during their early life and to improve lifetime traceability of horses bred for racing.^5^

For Thoroughbreds bred to compete in flat racing, the most prestigious and lucrative races are for 2‐ and 3‐year‐old horses^2^; that is, they occur in the third and fourth years of life. Therefore, breeders and owners aim for horses to enter training and race within this timeframe. In the current economic climate, where, even prior to the COVID‐19 pandemic, two‐thirds of UK Thoroughbred breeding operations were estimated to be unprofitable,^6^ premature losses from the industry or individuals failing to attain these milestones in a timely fashion could also negatively impact profitability, further threatening the industry's sustainability.

Prospective individual horse‐level follow‐up could provide up‐to‐date benchmarking and improved understanding around the fate of individuals failing to attain career milestones, as well as additional insight that could help to highlight traceability gaps and help inform strategies aimed at reducing wastage and improving sustainability. Through follow‐up of horses from an established birth cohort, the objectives of this study were therefore to: (1) describe proportions of Thoroughbreds bred for flat racing entering training and appearing on the racecourse, and (2) determine the horses’ fates and destinations, including any individuals not achieving these career milestones, by the end of the fourth year of life.

## MATERIALS AND METHODS

### Study design and period

The study design and recruitment strategy are described in detail elsewhere.^7^, ^8^ Briefly, a prospective cohort study was set up using a convenience sample of Thoroughbred stud farms across the UK and Ireland. All foals born on the recruited studs in 2019 entered the study and were under observation from birth until leaving the stud to enter training/pre‐training for racing at around 18 months of age, or prior to that, if they exited the study for another reason (e.g., death, being sold).^7^ To increase the sample size, some studs also agreed to enrol all foals born in 2020; these foals were under observation until 31 December 2020 or until exiting the study prior to that date.

For the study reported here, longer‐term follow‐up of the birth cohort horses included collection of all available sales, training, race performance, import and export, mortality, and location and usage data (details of registered owner/breeder/trainer/person responsible and current usage/status) to the end of the fourth year of life (i.e., until 31 December 2022 for those born in 2019 and 31 December 2023 for those born in 2020).

Sample size calculations were carried out using Epi Info (STATCALC version 7.2.6.0, Centers for Disease Control and Prevention, USA). It was estimated that a sample size of between 242 and 316 foals would be required to estimate a proportion of individuals entering training and racing of between 70% and 80%, with 95% confidence and 5% error in a population of 15,000 foals (estimated size of the combined UK and Ireland 2018 Thoroughbred foal crop^9^).

### Data collection

To describe the proportions of horses training and racing and the fates and destinations of those that failed to achieve these milestones, the main outcomes of interest were (1) whether horses had trained and raced anywhere in the world, and (2) their location and usage at the end of the follow‐up period, including any that had died. Therefore, follow‐up data, including dates of events, were collected for the following outcomes: named—name registered with stud book or horseracing authority or name as per sales catalogue or race start; sold—sold at any public auction worldwide, including date and location of auction, price achieved, details of vendor and purchaser; trained—details and date of registration with a licensed trainer worldwide and/or details of the trainer at entry of any race start worldwide; raced—record of any race start worldwide, including location and type of race, placing and prize money information; died—date of stud book or racing authority record of death or date that individual was reported by owner/breeder to have died; retired to stud—stud book authority record of broodmare or stallion registration or covering registered in return of mares/stud book authority database or record of having been sold certified (by a veterinarian) as being in foal or any records of having sired or produced progeny; exported—stud book or racing authority record of permanent export or record of appearing on a racetrack or any other event outside of the UK, for example, presented at an auction abroad without any evidence of subsequently returning to/appearing in the UK; and left industry—as per British Horseracing Authority records as reported by the trainer, including the given description of destination.

Data collection for the longer‐term follow‐up was undertaken via a number of data sources. First, intermittent email follow‐up was undertaken with participating stud farms about the fate and whereabouts of individuals that remained under their original ownership. Second, all available Weatherbys’ General Studbook and British Horseracing Authority data for individuals from the birth cohort were retrieved via a non‐disclosure agreement. Weatherbys’ data included: date of birth; dam; sire; sex; date of foal registration; naming and given name; date of death; last available date of export and import, along with the destination and whether this was permanent or temporary; sales data, including the name and date of the auction and the price/result of presentation at the auction; and date of registration as a broodmare or stallion. British Horseracing Authority data included: date of birth; dam; sire; sex; name; details of registration with trainers licensed in Great Britain, including date of registration and the name of the trainer; details of all race starts made in Great Britain and any foreign starts made against one or more Great Britain‐trained horse, including the date, racecourse, race type, placing and prize money won; and details of any individuals registered as having left the industry, including date of exit and description of destination, up to 31 December 2022 for those born in 2019 and 2023 for those born in 2020.

Additionally, all individuals known to be alive at the end of their second year of life and their respective dams were manually searched for in publicly available data sources,^10^, ^11^, ^12^, ^13^, ^14^, ^15^, ^16^, ^17^, ^18^, ^19^, ^20^, ^21^, ^22^, ^23^ from which any additional outcome data were collected. The data were collated and entered into the custom‐built Access (Microsoft Access for Microsoft 365, version 2208) database that was used for the birth cohort study.^7^, ^8^

### Data processing and analyses

All data were imported into Stata (Release 16, StataCorp). The data were described at the horse level. Age at the time of specific events, for example, death, sale or permanent export, was calculated by subtracting the date of birth from the date of the respective event, to allow for the evaluation of the distribution of events by age.

To better understand the population dynamics of the cohort, all recorded outcome events, that is, trained, named, raced, exported, sold, died, retired to stud and left industry, were sorted by horse and date. A flow diagram was constructed for the cohort from the end of the second year of life to the end of the fourth year of life, reporting events, fates and destinations, where available.

To describe available data and descriptive features of the cohort, histograms of continuous data were plotted and visually inspected for normality. The means and standard deviations (SDs) are reported for normally distributed data, and the medians and interquartile ranges (IQRs) are reported for non‐normally distributed data. Proportions and 95% confidence intervals (CIs) were calculated for the outcomes of interest for the cohort as a whole, by year and month of birth, and by farm. Differences in proportions between years, months of birth and farm were assessed using chi‐squared or Fisher's exact tests, with statistical significance set at a *p*‐value of less than 0.05.

## RESULTS

**FIGURE 1:**
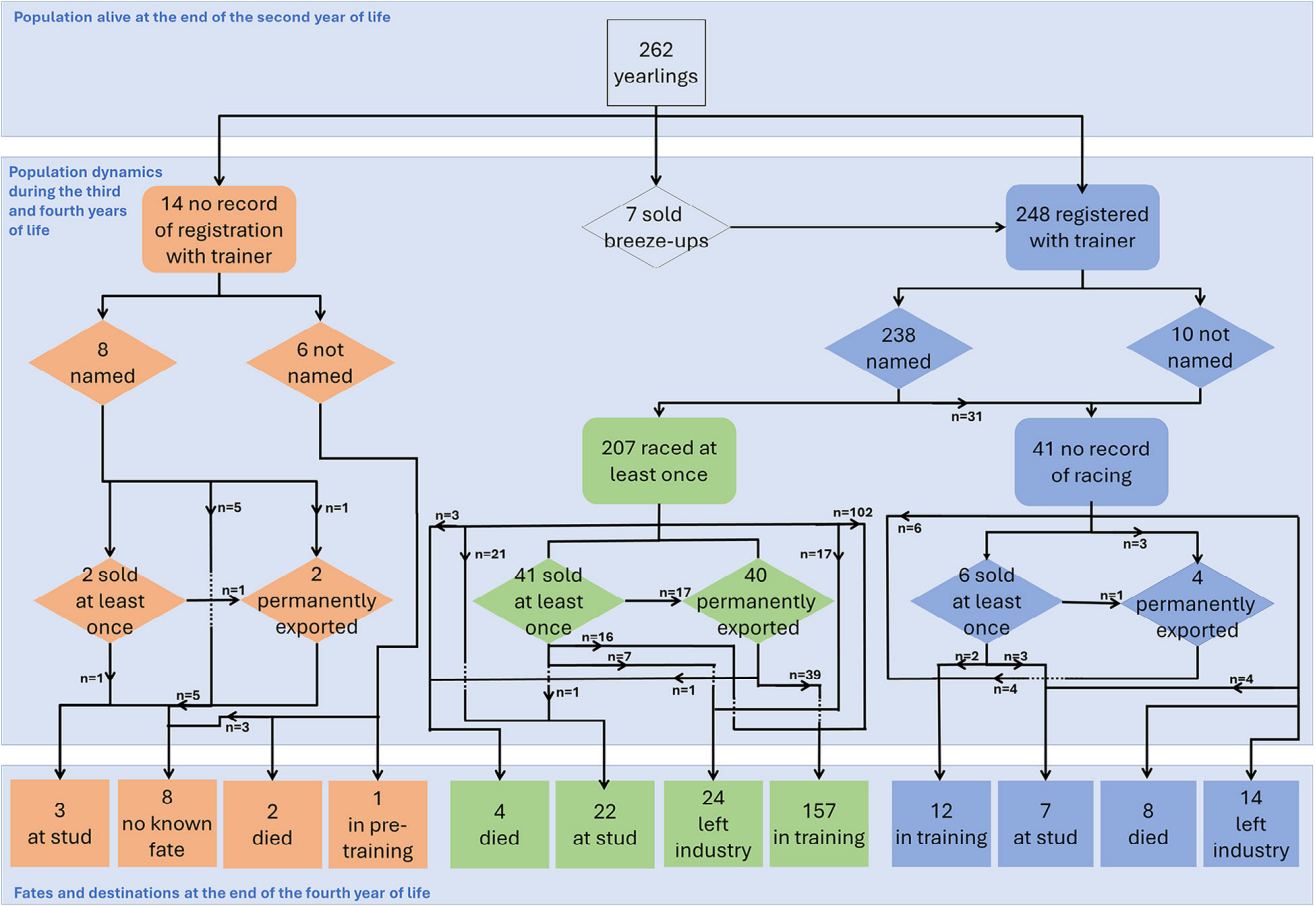
Population dynamics, fates and destinations of 262 Thoroughbreds, born in 2019 and 2020 across the UK and Ireland, between 31 December of the second year of life and 31 December of the fourth year of life. Breeze‐ups, unraced 2 year olds that are ridden and galloped –‘breezed’

A total of 275 foals (138 colts and 137 fillies) born over two seasons (197 in 2019 and 78 in 2020) on seven stud farms across the UK and Ireland were recruited to the initial birth cohort. Foals were born to 235 mares (40 of which foaled in both seasons), covered by 89 stallions. The average number of foals per farm was 39 (SD 39, range 13–135). Thirteen foals died before reaching 2 years of age,^7^ leaving a cohort of 262 yearlings (130 colts and 132 fillies) alive as of 31 December of the second year of life (2020 for those born in 2019 and 2021 for those born in 2020), for which to collect all available follow‐up outcomes to the end of the fourth year of life. Figure 1 presents an overview of the population dynamics of the cohort of 262 yearlings, including fates and destinations by the end of the fourth year of life.

### Training and racing

Of the population alive at the end of their second year of life, 94.7% (248/262; 95% CI 91.2‒96.8) entered training and 79.0% (207/262; 95% CI 73.7‒83.5) raced at least once, anywhere in the world, by the end of the fourth year of life, representing 90.2% (248/275; 95% CI 86.1‒93.2) and 75.3% (207/275; 95% CI 69.8‒80.0) of the original birth cohort, respectively. There were no significant differences (*p* > 0.05) in the proportions of horses entering training or racing between year or month of birth, or between stud farms.

The distribution of the geographical location of the first registered trainer for horses that entered training is presented in Table 1. Around two‐thirds of horses entered training in the UK, with the remainder spread across Europe, America, the Middle East, Japan and Australia.

**TABLE 1:** Distribution of the location of the first registered trainer for 248 Thoroughbred horses, born in 2019 and 2020 in the UK and Ireland, between birth and 31 December of the fourth year of life

Location of first registered trainer	Number of horses	Proportion of horses (%)	95% confidence interval (%)
UK	167	67.3	61.3‒72.8
France	25	10.1	6.9‒14.5
Ireland	23	9.3	6.2‒13.5
USA	9	3.6	1.9‒6.7
Germany	8	3.2	1.6‒6.2
Australia	3	1.2	0.4‒3.5
Italy	3	1.2	0.4‒3.5
Poland	3	1.2	0.4‒3.5
Czech Republic	2	0.8	0.2‒2.9
Hungary	2	0.8	0.2‒2.9
Bahrain	1	0.4	0.1‒2.2
Spain	1	0.4	0.1‒2.2
Japan	1	0.4	0.1‒2.2
			

### Mortality

Overall, 27 individuals died or were euthanased between birth and the end of the fourth year of life, representing an overall mortality of 9.8% (27/275; 95% CI 6.8‒13.9). Mortality was 4.7% (13/275; 95% CI 3.0‒7.9) in the first 2 years of life and 5.3% (14/262; 95% CI 3.2‒8.8) in the third and fourth years of life.

**FIGURE 2:**
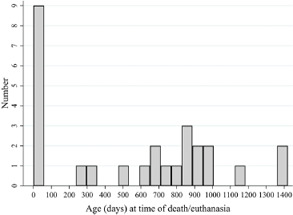
Distribution of age at time of death or euthanasia for a cohort of 275 foals, born on seven farms across the UK and Ireland in 2019 and 2020, between birth and 31 December of the fourth year of life

Figure 2 presents the distribution of age at death or euthanasia for all reported deaths from the cohort between birth and the end of the fourth year of life. The number of reported deaths was highest in the first 2 months of life, during which nine (3.3%; 9/275; 95% CI 1.7‒6.1) foals died or were euthanased.

### Sales

Overall, over the 4‐year study period, 67.3% (185/275; 95% CI 61.5‒72.5) of the cohort were sold at least once. There was a total of 242 sales transactions recorded for the cohort, with the maximum number of transactions per horse being 3 (median 1, IQR 0‒1). Overall, 11.2% of the cohort (31/275; 95% CI 8.0‒15.5) were sold as foals, 55.6% (153/275; 95% CI 49.7‒61.3) as yearlings, 2.5% (7/275; 95% CI 1.2‒5.2) as breeze‐ups (unraced 2 year olds that are ridden and galloped—‘breezed’) and 18.5% (51/275; 95% CI 14.4‒23.6) as horses in/out of training or breeding stock.

### Export

Overall, 29.8% (82/275; 95% CI 24.7‒35.5) of horses were recorded as having been permanently exported from the UK with no record of subsequent import by the end of the fourth year of life. Of these, 12.2% (10/82; 95% CI 6.8‒21.1) were exported as foals, 32.9% (27/82; 95% CI 23.7‒43.7) as yearlings, 12.2% (10/82; 95% CI 6.8‒21.1) as 2‐year olds and 42.7% (35/82; 95% CI 32.5‒53.5) as 3‐year olds. The distribution of reported destination countries is provided in Table 2.

**TABLE 2:** Distribution of reported export destinations for 82 horses, from a cohort of 275 Thoroughbreds born in 2019 and 2020 in the UK and Ireland, with a Weatherbys General Studbook record of permanent export from the UK between birth and 31 December of the fourth year of life

Export destination	Number of horses	Proportion of horses (%)	95% confidence interval (%)
USA	15	18.3	11.4‒28.0
France	10	12.2	6.8‒21.0
Australia	9	11.0	5.9‒19.6
Germany	9	11.0	5.9‒19.6
Italy	8	9.8	5.0‒18.1
Saudi Arabia	7	8.5	4.2‒16.6
United Arab Emirates	5	6.1	2.6‒13.5
Poland	3	3.7	1.2‒10.2
Hong Kong	3	3.7	0.7‒8.5
Bahrain	2	2.4	0.7‒8.5
Czech Republic	2	2.4	0.7‒8.5
Hungary	2	2.4	0.7‒8.5
Qatar	2	2.4	0.7‒8.5
Serbia	2	2.4	0.7‒8.5
Spain	1	1.2	0.2‒6.6
Sweden	1	1.2	0.2‒6.6
Portugal	1	1.2	0.2‒6.6
			

### Industry exit

Overall, according to British Horseracing Authority records, 15.3% (38/248; 95% CI 11.4‒20.2) of horses that had been registered with a Great Britain trainer were recorded as having left the industry during the study period, representing 13.8% (38/275; 95% CI 10.2‒18.4) of the original birth cohort. Of those reported by the British Horseracing Authority as having left the industry, 23 first destinations were recorded as being ‘unknown’ and 15 were recorded as ‘other, sports/leisure’.

### Retirement to stud

Overall, 11.6% (32/275; 95% CI 8.4‒16.2) of the birth cohort had records of having retired to stud by the end of the fourth year of life. Of these, 90.6% (29/32; 95% CI 75.8‒96.8) were in the UK or Ireland, and 9.4% (3/32; 95% CI 3.2‒24.2) were reported as having been permanently exported to Australia, France or Germany.

### Individuals with no recorded fate or destination

All of the eight (2.9% of the birth cohort; 8/275; 95% CI 1.5‒5.7) individuals (seven colts and one filly) with no recorded outcome after the end of the second year of life were sold at least once as either foals and/or yearlings. Five were also registered as having been named, of which three were recorded as having been permanently exported from the UK (one as a foal to Italy and two as yearlings to France and the Czech Republic, respectively). The three individuals (all colts) with no record of naming or permanent export had no further data touch points following sale as a foal, with their original stud farms/owners having no knowledge of their fate or whereabouts beyond their point of sale and no further records found in any industry or publicly available database.

## DISCUSSION

This study provides up‐to‐date benchmarking of the population dynamics of UK Thoroughbreds bred for flat racing and novel estimates of attainment of career milestones in addition to racing and training, such as retirement to stud and industry exit. The proportions of horses entering training and racing were significantly higher than previous estimates and industry‐level figures suggest, highlighting important traceability gaps at the industry level, most likely due to the dynamic nature of this population.

### Training and racing

Estimates of proportions of individuals entering training (95% of the cohort alive at the end of the second year of life and 90% of the total live‐foal crop) from this study are significantly higher than historical UK estimates. Analysis of the 1975 foal crop data estimated that 62% of horses named and eligible for training entered training by the end of the fifth year of life,^3^ and a study of 1022 Thoroughbreds born in 1999 estimated that 55% of the live‐foal crop entered training by the end of the fourth year of life.^2^ This suggests that wastage (i.e., non‐attainment of this milestone) has reduced over the last four decades, most likely due to advances in veterinary science,^24^ alongside increased investment in breeding and training facilities.^25^ Over the last two decades, there have also been important shifts in the demographics of UK Thoroughbred producers, where, due to a lack of profitability, many small‐ and medium‐sized breeders have left the industry.^6^, ^26^ These economic drivers, coupled with the recent COVID‐19 pandemic and an increased industry focus on sustainability,^27^ are believed to have not only impacted population demography but also size, with reductions in the broodmare band and foal crops and the number of UK‐based stallions,^9^ which in turn may have influenced wastage in this population.

The estimates obtained in this study are also significantly higher than those from a more recent analysis of UK industry data, which found that just 47% of the 2014 and 2015 UK and Ireland live‐foal crops had entered training by the end of the fourth year of life.^1^ It must, of course, be acknowledged that, at the industry level, not all horses are bred for flat racing and therefore expected to achieve this milestone within the 4‐year study period. For example, a further 14% of the 2014 and 2015 crops had entered training by the end of the fifth year of life.^1^ Nonetheless, current findings provide evidence that traceability gaps are likely to exist at the industry level due to the dynamic nature of this population. In the present study, one‐third of horses were first registered with a trainer outside of the UK and would therefore not appear as having trained and raced in industry databases (unless they had either raced in Great Britain or raced abroad against Great Britain‐trained horses, as is the convention for the BHA's recording of race performance data). Previous studies did not take account of exported horses entering training abroad^1^, ^2^ and, therefore, may have underestimated the true proportion of their study populations that trained. Such limitations must be carefully considered in the context of evaluating non‐attainment of career milestones and potential wastage.

Proportions of individuals appearing on the racecourse in the present study were also high, with 83% of the cohort that entered training making at least one race start by the end of the fourth year of life. This is comparable with two previous UK studies from which comparisons can be drawn over similar timeframes, which estimated that 81%^3^ and 79%^1^ of the horses from the respective cohorts that entered training raced at least once. However, when evaluating the proportion of the cohort alive at the end of the second year of life, and therefore eligible to enter training, that raced, the 79% reported in the current study compares favourably to the proportions reported in previous UK studies (51% to the end of fifth year of life^3^ and 39% to the end of the fourth year of life^1^). This provides further evidence not only that proportions of individuals attaining career milestones, and in turn wastage, may have improved over the last four decades, but also that important industry‐level traceability gaps exist around the population of horses training and racing outside of the UK.

### Mortality

Overall mortality estimates in the present study are similar to those reported in previous comparable studies, although there are some notable differences in the distributions of mortality over the study periods.^1^, ^2^ Of the cohort of 1022 foals born in 1999, 8% were reported to have died by the end of the fourth year of life, 72% of which were reported to have died by 2 years of age.^2^ In the analysis of industry‐level outcomes of the 2014 and 2015 UK and Ireland foal crops, 7% of live‐born foals had died by the end of the fourth year of life, of which 38% were reported to have died by 2 years of age.^1^ In the present study, 10% of the 275 foals had died by the end of the fourth year of life, of which 48% had died by 2 years of age. It is interesting to note that the relative distribution of mortality by age appears to have changed between the study from over 20 years ago,^2^ where almost three‐quarters of reported deaths occurred during early life, and the present study, where just under half of reported deaths occurred during this period. It is recognised that, due to advancements and investments in veterinary care and preventive health programmes, rates of infectious neonatal disease have reduced over time in this population, which may have contributed to these differences in relative mortality distribution.^7^, ^28^, ^29^

However, it is also important to evaluate any such differences in the context of the dynamics of the study populations being compared, legislation in place at the time of the study and the follow‐up methods used in the study itself. For example, it is likely that mortality outcomes are underreported to Weatherbys’ General Stud Book (the UK Passport Issuing Authority) for individuals that died while residing outside of the UK; therefore, differences in export rates and distribution of export by age between these studies should also be considered. In addition, the 30‐day foal notification legislation, requiring owners to notify Weatherbys of the foal's birth within 30 days,^30^ only came into effect in the UK from 2019 onwards. Prior to this, owners had up to 6 months to register a foal. It is therefore likely that mortality reporting compliance may have been poor in cases of death/euthanasia occurring during the first 6 months of life in unregistered foals with no passport. In contrast to the analysis of the Weatherbys and BHA databases,^3^ both the present study and that of Wilsher et al.^2^ undertook individual horse‐level follow‐up during this period, reducing the risk of underreporting of early‐life mortality in these study populations.

### Sales and export

In the study by Wilsher et al.,^2^ 11% of the cohort were sold as foals and 38% as yearlings, compared to 11% sold as foals and 56% as yearlings in the present study. It is unknown how similar the two study populations were in terms of relative proportions of commercial and owner‒breeder enterprises; therefore, making direct comparisons should be undertaken with caution. At the industry level, in 2020, 1506 yearlings were catalogued at Tattersalls' October sale, the largest sale of its type in Europe, which was estimated to represent around 30% of the 2019 UK Thoroughbred foal crop.^9^, ^31^ Given that there are seven major yearling sales across the UK and Ireland, estimated to sell over 2500 lots between them,^32^ it is likely that a large proportion of the foal crop are currently sold as yearlings each year.

In this context, the dynamic nature of Thoroughbred populations might pose potential challenges to traceability. In the present study, the only traceability gaps occurred in individuals that were sold during early life. Despite notification of change of ownership to the Passport Issuing Authority being a legal requirement in the UK,^33^ this is not the case in other countries. It is also recognised that this legislation is challenging to enforce in the UK,^34^ particularly prior to horses either being registered with a trainer or as a broodmare or stallion, whereupon an owner must be identified before registration can occur. In response to this, an initiative is currently being trialled by the Thoroughbred Breeders Association to provide free of charge transfer of ownership at Thoroughbred foal sales to encourage updating of ownership details at the point of sale in this population to facilitate traceability.^35^

In the present study, 30% of the cohort were recorded as having been permanently exported from the UK, with yearlings and 3 year olds most frequently exported. Probably unsurprisingly, this figure is much higher than that reported by Jeffcott et al.,^3^ where just 11% of the 1975 foal crop were reported to have been exported by 4 years of age, but is similar to that reported by Wilsher et al.^2^ Among the 1022 foals born in 1999, 37% were reported to have been exported by 4 years of age. Comparisons with export estimates from the study by Arango‐Sabogal et al.^1^ are not appropriate, as they did not distinguish between permanent and temporary exports.

### Life after racing

The present study provides an up‐to‐date estimate of the proportion of individuals retiring to stud from a UK Thoroughbred population, with 12% of the cohort having retired to stud by the end of the fourth year of life. This is similar to the 14% reported by Wilsher et al.^2^ However, it must be considered that estimates from both studies may underestimate individuals retiring to stud outside the UK, given that around one‐third of both cohorts were exported abroad. In the majority of foreign jurisdictions, it is not a requirement for breeding stock to be registered with the UK's Stud Book Authority. Additionally, unlike the UK, where a Return of Mares^10^, ^11^ is published each year, many jurisdictions do not keep a publicly available register of breeding animals; thus, in general, it is only possible to identify individuals being used for breeding purposes once their progeny are sold at public auction or appear on a racecourse.

With the continued decline of prize money in British racing,^26^ breeding stock, in particular stallions, generally generate much larger returns for owners (through sales of either coverings and/or progeny) while at stud compared to their returns from racing.^6^, ^26^ Therefore, once an individual has performed sufficiently to become valuable as a breeding animal, they are generally retired to stud, which could explain why a considerable proportion achieve this milestone during the first 4 years of life. Individuals retiring to stud are, of course, retained by the industry, and the fact that these numbers remain stable over time is a positive finding.

In the current study, another population that is likely to be underrepresented is those leaving the industry to move to second careers, because these data are only recorded in industry databases for those individuals who exit the industry directly from training in the UK. In the present work, by the end of the fourth year of life, 14% of the cohort were reported to have exited the industry by UK trainers. However, it was not possible to determine the destinations of individuals that may have been exported abroad for purposes other than training and racing, or that may have exited training to a second career outside of the UK. In the study by Wilsher et al.,^2^ 20% of the horses that did not continue racing from 2 to 3 years of age were reported as being used as riding horses, which would represent just 2% of the original cohort. A further 2% that did not enter training at 2 years of age were reported as being ‘not intended for racing’, so could perhaps also be assumed to be involved in other career paths at this time.

### Traceability gaps and limitations

In the present work, just 3% of the original cohort had no recorded fate or destination at the end of their fourth year of life, all of which had been sold as foals and/or yearlings, and three of which were known to have been permanently exported from the UK. This figure is similar to that reported by Wilsher et al.,^2^ who were unable to trace 4% of their cohort of foals born in 1999, but considerably lower than the 29% of the 2014 and 2015 UK and Ireland foal crops that had no recorded fate or destination in the study by Arango‐Sabogal et al.^1^ This again highlights the benefits of individual horse‐level follow‐up compared to analyses of industry‐level figures.

As previously discussed, the main limitation of the present study is that outcomes for horses outside of the UK are likely to be underestimated, as direct access to official stud book and racing authority databases was not available for jurisdictions outside of the UK, meaning that only publicly available data sources were used. Additionally, recording and notification of death and euthanasia were likely to be better reported for horses in training in the UK; therefore, mortality may be underestimated in other populations.

## CONCLUSIONS

Wastage due to failure to train and race may be significantly lower than industry‐level figures suggest, most likely due to the dynamic nature of this population and the challenges associated with capturing career milestones and other fates and destinations of horses that have left the UK. Better integration of industry data with import and export, horse disposal, and even retraining and other equestrian sports databases could help to reduce traceability gaps and, in turn, perceived wastage, and help maintain the industry's social licence to operate. It would also be advisable for the industry's lifetime traceability initiative^5^ to clearly define the target population and clarify whether this is solely aimed at individuals residing in the UK and under their jurisdiction. This would establish a more specific and well‐defined denominator population, enabling more accurate estimates that are less likely to be misinterpreted or fuel negative narratives of wastage.

## AUTHOR CONTRIBUTIONS

*Substantial contributions to conception and design of the study*: Verheyen, Mouncey and de Mestre. *Acquisition of data*: Mouncey. *Analysis of data*: Mouncey. *Interpretation of data*: Mouncey and Verheyen. *Drafting the article*: Mouncey. *Revising it critically for important intellectual content*: Mouncey, de Mestre and Verheyen. *Final approval of the version to be published*: Mouncey, de Mestre and Verheyen. *Accountable for all aspects of the work*: Mouncey, de Mestre and Verheyen. The corresponding author confirms that she had full access to all the data in the study and takes responsibility for the integrity of the data and the accuracy of the data analysis.

## CONFLICT OF INTEREST STATEMENT

The authors declare they have no conflicts of interest.

## ETHICS STATEMENT

Ethical approval was granted by the Royal Veterinary College's Clinical Research Ethical Review Board (URN: 2024 2251‐2). Owner informed consent was obtained for enrolment of animals into this study. British Horseracing Authority and Weatherbys’ General Stud Book data were collected under a non‐disclosure agreement.

## Data Availability

The data that support the findings of this study are available from the corresponding author upon reasonable request.
